# Nutritional Composition and Bioactive Content of Legumes: Characterization of Pulses Frequently Consumed in France and Effect of the Cooking Method

**DOI:** 10.3390/nu10111668

**Published:** 2018-11-04

**Authors:** Marielle Margier, Stéphane Georgé, Noureddine Hafnaoui, Didier Remond, Marion Nowicki, Laure Du Chaffaut, Marie-Josèphe Amiot, Emmanuelle Reboul

**Affiliations:** 1 C2VN, INSERM, INRA, Aix-Marseille Univ, 13385 Marseille, France; Marielle.margier@univ-amu.fr (M.M.); Marion.Nowicki@univ-amu.fr (M.N.); 2 Biochemistry Department, Centre Technique de Conservation des Produits Agricoles (CTCPA), site Agroparc, 84911 Avignon, France; sgeorge@ctcpa.org; 3 UNH, INRA, CRNH Auvergne, Université Clermont-Auvergne, F-63000 Clermont-Ferrand, France; noureddine.hafnaoui@inra.fr (N.H.); didier.remond@inra.fr (D.R.); 4 ANSES, 94700 Maisons-Alfort, France; Laure.DUCHAFFAUT@anses.fr; 5 MOISA, Univ Montpellier, CIRAD, CIHEAM-IAMM, INRA, Montpellier SupAgro, 34000 Montpellier, France; Marie-Josephe.Amiot-Carlin@inra.fr

**Keywords:** proteins, essential amino acids, lipids, fibers, vitamins, carotenoids, minerals, saponins, phytates, tannins

## Abstract

Pulses display nutritional benefits and are recommended in sustainable diets. Indeed, they are rich in proteins and fibers, and can contain variable amounts of micronutrients. However, pulses also contain bioactive compounds such as phytates, saponins, or polyphenols/tannins that can exhibit ambivalent nutritional properties depending on their amount in the diet. We characterized the nutritional composition and bioactive compound content of five types of prepared pulses frequently consumed in France (kidney beans, white beans, chickpeas, brown and green lentils, flageolets), and specifically compared the effects of household cooking vs. canning on the composition of pulses that can be consumed one way or the other. The contents in macro-, micronutrients, and bioactive compounds highly varied from one pulse to another (i.e., 6.9 to 9.7 g/100 g of cooked product for proteins, 4.6 to 818.9 µg/100 g for lutein or 15.0 to 284.3 mg/100 g for polyphenols). The preparation method was a key factor governing pulse final nutritional composition in hydrophilic compounds, depending on pulse species. Canning led to a greater decrease in proteins, total dietary fibers, magnesium or phytate contents compared to household cooking (i.e., −30%, −44%, −33% and −38%, *p* < 0.05, respectively, in kidney beans). As canned pulses are easy to use for consumers, additional research is needed to improve their transformation process to further optimize their nutritional quality.

## 1. Introduction

Because pulses present both environmental and nutritional benefits, they are often recommended in sustainable diets [1]. Their environmental benefit is related to their ability to restore soil nitrogen without adding fertilizer. Diversifying crop rotations with pulses is thus a green solution to enhance system productivity [2]. Moreover, health organizations such as the Food and Agriculture Organization of the United Nations (FAO) recommend pulses as staple foods to fulfill the basic protein and energy requirements of the human diet [3]. Indeed, pulse grains are i) a low-fat source of proteins and carbohydrates and ii) of interest as a gluten-free food category. They exhibit complementary amino acid profiles to those of cereals in well-balanced semi-vegetarian or plant-based diets [4]. They are also rich in fibers and can contain variable amounts of other nutritional components such as vitamins, minerals, and bioactives [5]. This likely explains why significant consumption has been associated with improved lipid parameters [6,7,8] and colic function [9], as well as decreased risks of developing pathologies such as diabetes, cardiovascular or degenerative diseases [5]. However, some bioactive compounds present in pulses can display ambivalent properties [10]. It has been suggested that low doses of phytates could reduce the risk of colon cancer because of i) phytate antioxidant effect, and ii) phytate prebiotic activity due to their ability to bind enzymes such as amylases, so that a portion of the undigested starch reaches the intestine [11]. Conversely, at higher doses, phytates act by chelating different cations, thereby decreasing the bioavailability of the minerals present in the bolus [11]. Furthermore, it is acknowledged that saponins interfere with the absorption of dietary lipids, cholesterol, and bile acids - thus displaying interesting lipid-lowering properties, but also with vitamins A and E in chicks [12]. Saponins have additionally been shown to bind to the cells of the small intestine, which can affect the in vitro absorption of nutrients across the intestinal wall [13]. Finally, tannins, well known for their antioxidant capacity [5], have been found to interfere with the digestion by displaying anti-trypsin and anti-amylase activity [14,15]. They are likely responsible for the trypsin inhibitor activity of fava beans [16]. They have also been reported to interfere with iron and zinc absorption [17].

Cooking and thermal treatments in general usually cause losses in food product nutritional quality and phytochemical contents. However, they can also inactivate heat labile antinutritional factors such as pulse antitrypsin factors that adversely affect protein bioavailability [18], decrease pulse content in unwanted factors such as phytates [19], or modulate pulse amino acid composition and protein digestibility [20]. Most of the works conducted so far to characterize pulse nutritional composition have focused either on one pulse, or on limited nutrient, micronutrient, or bioactive categories. The present study thus aimed at comprehensively describing the overall nutritional value, including both nutrient (proteins and amino acids, fat, fibers, minerals, and vitamins) and bioactive compound (saponins, phytates, polyphenols, and tannins) profiles, of the main cooked pulses typically consumed in France (kidney beans, white beans, chickpeas, brown/green lentils, and flageolets). Pulses were prepared according to the way they are usually consumed. To respect traditional eating habits, green lentils and flageolets were thus prepared using household cooking only, while brown lentils were prepared using canning only. Kidney beans, white beans, and chickpeas were prepared using both methods, allowing us to specifically compare the effect of the preparation method on the pulse nutritional composition.

## 2. Materials and Methods

### 2.1. Materials

Kidney beans (*Phaseolus Vulgaris* L.), white beans (*Phaseolus Vulgaris* L.), chickpeas (*Cicer Arietinum*), green and brown lentils (*Lens culinaris* M.), and flageolets (*Phaseolus vulgaris* L.) were purchased from CIACAM (Vitrolles, France). Phytic acid, soyasaponin (*Soyasaponin βb*), vanillin, α-tocopherol, γ-tocopherol, and retinyl acetate were from Sigma Aldrich (Saint-Quentin-Fallavier, France). Pure carotenoids used for calibration curves were a generous gift from DSM (Basel, Switzerland).

For all quantification, ultrapure deionized water was purified by a Millipore ultrapure system to a specific resistance of 18 mΩ.cm or greater (SynergyWater Purification System, Millipore, Molsheim, France). All solvents were HPLC grade and from either Fisher Scientific (Illkirch-Graffenstaden, France) or Carlo Erba (Peypin, France).

### 2.2. Preparation Methods

All pulses were prepared immediately after purchase.

#### 2.2.1. Household Cooking Method

Household cooking was performed according to the protocols of the French National Federation of Dry Legumes (FNLS, Paris, France) as follows. Except for lentils, pulses were soaked in low ionized water (water hardness 10–15 °F) at a ratio of 1:5 (seed:water; *w*:*w*) overnight at room temperature. After draining the water, pulses were cooked in mineral water (Pyrenea, Auchan, Croix, France) at a ratio of 1:2 (seed:boiling water) for 25 min for lentils, 1 h 30 min for white beans, kidney beans and flageolets, and 2 h for chickpeas. Three independent cooking s of each pulse were performed.

#### 2.2.2. Canning Method

Canning was performed according to the protocols of the Technical Center of Food Product Conservation (CTCPA Food Technical Center, Avignon, France) and the seeds were industrially processed by the CTCPA. The seeds were soaked in low ionized water at a ratio of 1:3 (seed:water; *w*:*w*) overnight at room temperature. Afterwards, blanching was performed at 90 °C for 5 min. The seeds were then conditioned in a can (425 mL), and hot brine (0.5% salt, −70 °C) at a ratio of 195/236 (*w*/*w*) was added. The cans were sterilized at 127 °C for 16 min, and subsequently cooled to 30 °C for 10 min.

#### 2.2.3. Stabilization Method

The processed seeds were drained, rinsed in water, and frozen. Then, the cooked seeds were freeze-dried, milled into flour using a Pulverisette 2 mill (Fritsch, dar-Oberstein, France), and stored at −80 °C in plastic tubes until analysis. 

### 2.3. Nutritional Composition Analysis of Pulses

#### 2.3.1. Protein and Amino Acid Composition

Amino acid analysis was performed with an amino acid analyzer (L-8900, Hitachi, Paris, France), after four different protein hydrolysis: (i) acid hydrolysis with 6 N HCl for 24 h at 110 °C; (ii) acid hydrolysis with 6 N HCl for 48 h at 110 °C, for branched-chain amino acids; (iii) acid hydrolysis with 6 N HCl for 24 h at 110 °C, after performing acid oxidation, for sulfur amino acids; (iv) basic hydrolysis with 4 N Ba(OH)_2_ for 16 h at 110 °C, for tryptophan. For each hydrolysis, norleucine was used as the internal standard. Total protein content was estimated as the sum of all amino acid contents. 

#### 2.3.2. Lipid Composition

Pulse flour samples (500 mg) were homogenized in 800 µL of PBS. Lipids were first extracted following the Bligh and Dyer methods [21]. Concentration of total lipids were measured by weighing the dry extract, and then the samples were resuspended in isopropanol. Concentration of total sterol, phospholipid, and triacylglycerol from lipid extracts were measured using kits from Biolabo (Maisy, France) according to the manufacturer’s instructions.

#### 2.3.3. Fat-Soluble Micronutrient Composition

Vitamin D, vitamin E (α-tocopherol, γ-tocopherol), vitamin K (phylloquinone) and carotenoids (provitamin A = β-carotene, lutein, and zeaxanthin) were extracted from 500 mg of pulse flour using the following method: 2 mL of distilled water were added to sample. The internal standard (retinyl acetate) was added to the sample in 2 mL of ethanol. The mixture was extracted twice with 8 mL of hexane. After centrifugation (500× *g*, 10 min at 4 °C), 2 mL of distilled water and 2 mL of ethanol were added at the hexane phase. The hexane phase obtained after centrifugation (500× *g*, 10 min at 4 °C) was evaporated to dryness under nitrogen. The dried extract was dissolved in 200 µL of methanol–dichloromethane (65/35, *v*/*v*). A final volume of 150 µL for samples was used for HPLC analysis. The fat-soluble vitamins and carotenoids were separated as previously described [22,23,24]. All molecules were identified by retention time compared with pure standards.

#### 2.3.4. Analysis of Bioactive Compounds

Phytates were analyzed by spectrometric method derived from the method published by Dost and Tokul [25]. Briefly, phytates were extracted from flour seeds with chloridric acid (0.5 M, Fisher Scientific, Saint Herblain, France) for 1 h at room temperature. Then the extract was centrifuged (10 min at 800× *g*) and the supernatant was recovered. Next 0.1 mL of supernatant was added to 0.9 mL of water and 2 mL of iron (III) chloride hexahydrate (Acros Organics, Noisy le Grand, France). This mixture was mixed for 2 h 30 min at 40 °C and then centrifuged (5 min at 800× *g*). The supernatant was spectrometrically measured at 480 nm against water.

Saponins were analyzed by a spectrometric method inspired by the method previously published by Cheok et al. [26]. The first step was the extraction of saponins from the flour seed matrix. One milligram of flour was added to 5 mL of methanol (80% in water), mixed for 24 h under room temperature and then centrifuged (5 min at 800× *g*). Supernatants (0.2 mL) were completed with 0.3 mL of methanol (80% in water), 0.5 mL of vanillin (8% in water), and 5 mL of sulfuric acid (72%), and the mixtures were incubated at 60 °C for 10 min then placed into an ice bath. Mixtures were spectrometrically measured at 544 nm against methanol (80% in water).

Total polyphenols were analyzed following the method developed by Georgé et al. (2005) [27]. Tannins were quantified using the DMACA (*p-*dimethylaminocinnamaldehyde) reagent. Two milliliters of a water/methanol mixture (1/1; *v*/*v*), 4 mL of polyphenol extract, and 1 mL of DMACA solution were mixed together. After 20 min, the absorbance was measured between 638 nm and 643 nm against a water/methanol mixture (1/1). The results were expressed as the catechin equivalent.

#### 2.3.5. Total Dietary Fiber, Water-Soluble Vitamin and Mineral Contents

These analyses were subcontracted to Eurofins analytics laboratory (Nantes, France), which is specifically accredited for these analyses (cofrac #1-0287).

### 2.4. Statistical Analysis

Analyses were performed in triplicate and data are presented as mean ± standard error. Differences between more than two groups of unpaired data were tested by the Kruskal‒Wallis test followed by the Mann‒Whitney U-test, used as a post hoc test. Differences between only two groups of unpaired data were tested by the Mann‒Whitney U test. Values of *p* < 0.05 were considered significant. All statistical analyses were performed using Statview software, version 5.0 (SAS Institute, Cary, NC, USA).

## 3. Results

### 3.1. Proteins

As shown in Table 1, household-cooked kidney beans were the richest in protein (9.7 g/100 g), while canned brown lentils were the poorest (5.1 g/100 g). Individual amino acid levels are reported in Table 2. Pulses were well balanced between essential and non-essential amino acids (45% and 55%, respectively, Table 1). The predominant essential amino acid were leucine, lysine, and valine (i.e., 0.785 g/100 g for Leu, 0.670 g /100 g for Lys, and 0.512 g/100 g for Val in household-cooked kidney beans). As expected, pulses were very low sources of essential sulfur amino acids (Cys and Met). In all pulses, the major non-essential amino acids were asparagine + aspartic acid (Asp) and glutamine + glutamic acid (Glu) (i.e., 1.17 and 1.55 g/100 g for household-cooked kidney beans).

Canned kidney beans, white beans, and chickpeas had a significantly lower protein content (−30%, −18%, and −17%, respectively; *p* < 0.05) than paired household-cooked pulses. Kidney beans displayed a major reduction in both essential and non-essential amino acids (−29% and −32%). This reduction was less important in chickpeas (−16% and −18%). In white beans, the reduction of essential amino acids was stronger than the reduction of non-essential amino acids (−27% vs. −10%, *p* < 0.05).

### 3.2. Lipids

Although pulses are relatively poor in lipids, high variability could be observed from one pulse to another. Household-cooked chickpeas had a higher total lipid content than the other pulses (4- to 9-fold whatever the preparation method, *p* < 0.05, Table 1). This was due to the fact that sterol and phospholipid contents were 4- to 10-fold higher in chickpeas than in other pulses (*p* < 0.05).

Canning led to an increase in both the sterol and triglyceride contents of kidney beans (+34% and +24%, respectively; *p* < 0.05), but decreased the phospholipid content of white beans and chickpeas (−33% and −6%, respectively; *p* < 0.05) and the triglyceride content of chickpeas (−12%; *p* < 0.05) compared with household cooking. Overall, canning moderately but significantly decreased the total lipid content of chickpeas (−13%), but had no effect on the total lipid content of other pulses.

### 3.3. Total Dietary Fibers (TDF)

Our results highlighted that TDF constitute a major component of cooked pulses (from 13.8 g/100 g for household-cooked flageolets to 3.8 g/100 g for canned brown lentils). 

The canning process caused a significant decrease in TDF level compared to the paired household-cooked pulses (−44% for kidney beans, −33% for white beans, and −22% for chickpeas; *p* < 0.05).

### 3.4. Fat-Soluble Micronutrients

The studied pulses were not particularly rich in fat-soluble vitamins. Indeed, vitamin D, K, and α-tocopherol were not detected in any pulses (data not shown). Furthermore, our results showed a great variability in γ-tocopherol and carotenoid (β-carotene, lutein, and zeaxanthin) contents among the studied pulses. Among carotenoids, lutein was predominant in all pulses. Lentils were the richest pulses in γ-tocopherol (4.43 mg/100 g in household-cooked green lentils and 3.51 mg/100 g in canned brown lentils), and also displayed the highest content of lutein (748 µg/100 g in household-cooked green lentils and 818 µg/100 g in canned brown lentils). 

Finally, canning had a positive effect on the γ-tocopherol content in kidney beans compared to household cooking (+135%; *p* < 0.05) and on the lutein content in kidney beans and in chickpeas (+22% and +74%, respectively).

### 3.5. Water-Soluble Vitamins

Table 1 presents the main soluble vitamin (B1, B2, B5, B6, and B9) contents. Vitamin C was not detected in pulse samples. The contents in vitamin B1 and B2 were 2- to 14-fold higher in kidney beans than in the other pulses (*p* < 0.05). After household cooking, green lentils and chickpeas displayed the higher contents in vitamin B5 and B6 compared to other pulses (*p* < 0.05). Finally, vitamin B9 content was 2- to 4-fold higher in chickpeas than in the other pulses (*p* < 0.05).

The cooking method had no effect on vitamin B1, B5, and B6 contents, while vitamin B2 was strongly reduced by the canning process. Compared to household cooking, canning also led to a significant reduction in vitamin B9 content in white beans and chickpeas (−57.9% and −49.3%, respectively; *p* < 0.05).

### 3.6. Minerals

In all pulses, the major minerals were potassium (from 81 to 300 mg/100 g), phosphorus (from 53 to 150 mg/100 g), calcium (from 27 to 120 mg/100 g), and magnesium (from 13 to 44 mg/100 g). Iron and zinc were present in small quantities ranging from 1.3 to 2.3 mg/100 g for iron and 0.48 to 1.10 mg/100 g for zinc, respectively. Neither selenium nor iodine were detected in our samples. Note that the sodium content was not evaluated because, conversely to household cooking, the canning process requires the addition of salt.

Comparable values for iron and zinc were observed between household-cooked and canned white beans and chickpeas. A loss of 21.7% was observed for iron in canned kidney beans compared to household-cooked kidney beans. A loss of 33.3% and 36.4% was also observed for magnesium in canned kidney beans and chickpeas compared to paired household-cooked pulses. 

### 3.7. Bioactive Compounds

Phytates were abundant in all pulses, assayed from 413 mg/100 g in brown lentils to 714 mg/100 g in green lentils. Saponin contents were around 120 mg/100 g (mean value). Great variations were seen for tannins (from 1.7 mg/100 g in household-cooked white beans to 16.6 mg/100 g in household-cooked chickpeas) and total polyphenols (from 15 mg/100 g in household-cooked white beans to 284 mg/100 g in household-cooked green lentils).

The method of preparation could significantly influence pulse bioactive compound composition. For instance, phytate contents were lower in canned kidney beans and chickpeas than in paired household-cooked grains (−38.5% and −24%, *p* < 0.05). Conversely, polyphenol content was higher in canned white beans and chickpeas compared to household-cooked white beans and chickpeas (+24.4% and +10.5%, respectively, *p* < 0.05). The saponin content moderately varied depending on the preparation method: it was significantly higher in canned kidney beans than in household-cooked kidney beans (+12%), whereas it was significantly lower in canned chickpeas compared to household-cooked chickpeas (−4.1%).

## 4. Discussion

The first objective of our study was the assessment of the overall nutritional composition of different prepared pulses regularly consumed in France. Raw pulses were purchased from CIACAM, one of the leading companies on the French pulse market, and thus represent the grains available from French processing companies. Pulses were prepared according to standard protocols provided by the French National Federation of Dry Legumes and the Technical Center of Food Product Conservation. We confirmed that the pulses displayed a good nutritional profile in terms of nutritional density. Indeed, both canned and household-cooked pulses were high in proteins and fibers and low in fat. They were not particularly rich in vitamins but contained interesting amounts of oligoelements according to previous data [5].

Proteins represented 19‒28% of the dry matter. Although the sulfur amino acid content remained low, which confirms that pulse proteins are complementary to cereal proteins [28], the studied pulses were rich in essential amino acids. Indeed, the contribution of 200 g of household-cooked kidney beans to the recommended amino acid intakes for a 70 kg man [29] is 72% for His, 72% for Ile, 57% for Leu, 63% for Lys, 81% for Thr, 78% for Trp, and 81% for Val. This good coverage of essential amino acids explains how pulses can partially substitute for animal proteins in a vegetarian diet [4].

Pulse total dietary fiber contents represented up to 41% of dry matter for household-cooked flageolets. We showed that chickpea and lentil fiber contents were 21.5 and 24.8 g/100 g of dry matter, respectively. These data are consistent with a previous report showing that soaked and cooked chickpea and lentil fiber contents were 24.7 and 30.4 g/100 g of dry matter, respectively [30].

Conversely, lipids represented less than 5.4% of the dry matter. The maximum lipid level was 2.0 g/100 g in household-cooked chickpeas, while it can reach 10 g/100 g in cooked red meat or 19.9 g/100 g in soy beans [31]. All pulses were particularly low in sterols.

Chickpeas and lentils contained some vitamin E (γ-tocopherol), which could be of interest because a significant proportion of the population does not achieve the recommended vitamin E intake in both Europe and the USA [32]. Interestingly, pulses and especially brown and green lentils contained both lutein and zeaxanthin (0.87 and 0.79 mg/100 g, respectively). These fat-soluble plant-derived microconstituents likely participate in the prevention of eye diseases including age-related macular degeneration [33]. The studied pulses also presented significant amounts of oligoelements such as copper, iron, magnesium, manganese, and zinc, in comparable amounts to those previously reported [34].

Finally, our results highlight that all prepared pulses were a significant source of saponins, phytates, and tannins: 99 to 175 mg saponins/100 g, 380 to 694 mg phytates/100 g, and 15 to 284 mg of polyphenols/100 g; the richest in these molecules were green lentils, canned white beans, and flageolets. Pulses’ high tannin and phytate contents may constitute a nutritional issue in unbalanced diets, providing reduced quantities of minerals such as iron or zinc. Methods to reduce pulse phytate content such as soaking, germination, or sourdough fermentation are thus recommended, especially in vegetarian diets [35].

The second objective of this work was to specifically compare the effect of a traditional cooking method (soaking and cooking in water) or canning (soaking, blanching, and a simultaneous process of canning/cooking/autoclaving) on kidney bean, white bean, and chickpea nutritional profiles. As the same raw pulses were used to prepare both canned and household-cooked pulses, the differences in nutritional composition observed between drained canned and drained household-cooked pulses are only due to the preparation process.

Canning caused a significant decrease in grain protein and fiber contents (up to −30% and −44%, respectively) compared to household cooking. We suggest that canning led to a higher matrix disruption than household cooking, which induced increased leaching of both proteins and fibers to canning broth. Similarly, canning induced a higher negative impact on water-soluble micronutrient content than household cooking, likely because leaching, heating, and oxidation reactions were more pronounced during this cooking method. Indeed, water-soluble vitamins are known to be eliminated during both the soaking and cooking stages [36]. 

Interestingly, the cooking method had a minor effect on pulse mineral contents. We found a small decrease or no decrease in potassium, calcium, phosphorus, copper, or zinc contents in canned pulses compared to paired household-cooked grains. For instance, in household-cooked vs. canned white beans, the mineral contents were as follows: potassium = 230 vs. 230 mg/100 g; calcium = 79 vs. 77 mg/100 g; phosphorus = 120 vs. 110 mg/100 g; copper = 0.22 vs. 0.26 mg/100 g, iron = 1.6 vs. 1.5 mg/100 g, and zinc = 0.76 vs. 0.74 mg/100 g, respectively. These results are in accordance with those previously published by Rickman et al. [37]. This could be explained by the formation of complexes between divalent cations and catechol-bearing molecules (polyphenols, especially tannins) or phytates, which can limit their transfer into the canning water during the thermal treatment [38].

Our data also highlight that canning allowed a higher elimination of some bioactive compounds compared to household cooking such as phytates (a mean reduction of 25%). This effect can be explained by more efficient leaching during processing combined with an activation of endogenous phytases during soaking [30,39]. Regarding tannin contents, we found no difference between the two cooking practices, in agreement with Wang and colleagues [39].

Overall, the reduction in water-soluble compound contents in pulses after canning was in agreement with that reported in the literature for processed fruits, nuts, and vegetables. These losses can be attributed to the extensive heat exposure during thermal processing or to the leaching of some of the compound into the broth due to the softening of the cell walls that occurs during cooking [40,41,42].

Interestingly, canning led to a greater recovery of fat-soluble micronutrient contents compared to household cooking. This is likely due to a better extractability of fat-soluble compounds from a more disrupted matrix [37,43,44].

## 5. Conclusions

A vital challenge today is to change our current diets to healthier and more sustainable diets. In this context, the promotion of pulse consumption is widely acknowledged and promoted. However, a first limitation to pulse consumption is related to their cooking process. Uncooked dry pulses can be perceived as challenging to prepare [45]. Indeed, the traditional preparation generally requires two steps: soaking (between 18 and 24 h) and cooking (between 15 min and 2 h at 85–95 °C) [19]. In comparison, the use of processed pulses such as canned pulses appears to be a convenient and affordable way to consume legumes [46]. A second limitation is linked to pulse consumption in an unbalanced diet. In a well-balanced diet, pulse bioactive compounds may contribute to reduce the risk of chronic diseases [47]. However, in an unbalanced diet, compounds such as phytates may decrease the nutritional quality of the diet by interfering with mineral bioavailability [11]. Interestingly, canning appears to be beneficial to limit the phytate content of pulses compared to household cooking. Additional research is thus needed to limit the concomitant losses in nutrients and micronutrients also observed during the canning process. To this end, a comprehensive assessment of the nutritional composition of both pulses and soaking water/broth at different stages of the soaking and the canning process would provide further useful information.

## Figures and Tables

**Table 1:** Nutritional and bioactive compound compositions of prepared kidney beans, white beans, chickpeas, green and brown lentils, and flageolets.

Pulses	Kidney Beans		White Beans		Chickpeas		Green Lentils	Brown Lentils	Flageolets
Cooking Method	Household Cooking	Canning	Household Cooking	Canning	Household Cooking	Canning	Household Cooking	Canning	Household Cooking
Proteins (g/100 g)	9.67 ± 0.10 ^a^	6.74 ± 0.14 ^f,^*	7.20 ± 0.08 ^b^	5.92 ± 0.12 ^g,^*	7.82 ± 0.08 ^c^	6.46 ± 0.13 ^h,^*	8.31 ± 0.09 ^d^	5.08 ± 0.10 ^i^	6.92 ± 0.07 ^e^
Essential AA	4.47	3.18	3.42	2.51	3.45	2.89	3.43	2.15	3.33
Non-essential AA	5.20	3.56	3.79	3.41	4.37	3.57	4.88	2.93	3.59
Lipids									
Total lipids (g/100 g)	0.28 ± 0.03 ^a^	0.29 ± 0.01 ^f^	0.26 ± 0.01 ^a^	0.23 ± 0.03 ^g^	2.04 ± 0.03 ^b^	1.78 ± 0.06 ^h,^*	0.29 ± 0.01 ^a^	0.19 ± 0.01 ^i^	0.51 ± 0.03 ^c^
Triglycerides (g/100 g)	0.16 ± 0.01 ^a^	0.20 ± 0.01 ^f,^*	0.21 ± 0.01 ^b^	0.19 ± 0.03 ^f^	1.92 ± 0.00 ^c^	1.69 ± 0.03 ^g,^*	0.16 ± 0.02 ^a^	0.14 ± 0.00 ^h^	0.44 ± 0.04 ^d^
Phospholipids (mg/100 g)	20.84 ± 0.41 ^a^	20.94 ± 2.53 ^f^	17.98 ± 0.95 ^b^	11.97 ± 3.36 ^g,^*	165.85 ± 0.65 ^c^	156.09 ± 5.38 ^h,^*	23.96 ± 0.76 ^d^	17.90 ± 1.44 ^f^	39.31 ± 4.28 ^e^
Sterols (mg/100 g)	22.05 ± 1.23 ^a^	29.62 ± 1.23 ^f,^*	29.21 ± 0.77 ^b^	28.25 ± 2.88 ^f^	114.99 ± 12.88 ^c^	121.02 ± 9.26 ^g^	29.15 ± 3.93 ^b^	19.16 ± 0.30 ^h^	43.25 ± 0.81 ^d^
Total dietary fibers (g/100 g)	11.60 ± 2.50 ^ab^	6.50 ± 2.10 ^f,^*	10.00 ± 2.40 ^ab^	6.70 ± 2.20 ^f^	8.20 ± 2.30 ^a^	6.40 ± 2.10 ^f^	8.50 ± 2.30 ^a^	3.80 ± 1.90 ^f^	13.80 ± 2.70 ^a^
Fat-soluble micronutrients									
γ-tocopherol (mg/100 g)	0.43 ± 0.21 ^a^	1.02 ± 0.23 ^f,^*	n.d.	n.d.	2.20 ± 0.40 ^b^	2.88 ± 0.64 ^g^	4.43 ± 0.40 ^c^	3.51 ± 0.63 ^g^	0.27 ± 0.10 ^a^
β-carotene (µg/100 g)	n.d.	n.d.	n.d.	n.d.	14.56 ± 2.95 ^a^	14.69 ± 2.59 ^f^	14.34 ± 1.91 ^a^	6.87 ± 0.71 ^g^	n.d.
Lutein (µg/100 g)	4.62 ± 1.02 ^a^	5.65 ± 0.61 ^f^	n.d.	n.d.	106.65 ± 53.58 ^b^	185.86 ± 62.76 ^g^	748.76 ± 76.03 ^c^	818.90 ± 110.02 ^h^	7.48 ± 1.84 ^d^
Zeaxanthin (µg/100 g)	0.78 ± 0.18 ^a^	0.86 ± 0.10 ^f^	n.d.	n.d.	10.19 ± 5.12 ^b^	11.21 ± 1.35 ^g^	45.89 ± 4.92 ^c^	49.41 ± 6.76 ^h^	n.d.
Water-soluble vitamins (µg/100 g)									
B1	117.00 ± 19.00 ^a^	90.00 ± 14.40 ^f^	23.00 ± 3.70 ^b^	20.00 ± 3.20 ^g^	60.00 ± 9.60 ^c^	50.00 ± 8.00 ^h^	72.00 ± 11.50 ^c^	40.00 ± 6.40 ^i^	30.00 ± 4.80 ^b^
B2	220.00 ± 3.50 ^a^	n.d.	15.80 ± 2.50 ^b^	n.d.	26.80 ± 4.30 ^c^	n.d.	32.70 ± 5.20 ^c^	n.d.	16.80 ± 2.70 ^b^
B5	83.30 ± 20.00 ^a^	90.40 ± 21.70 ^fg^	72.70 ± 17.40 ^a^	74.40 ± 17.90 ^g^	138.00 ± 33.00 ^b^	146.00 ± 35.00 ^fh^	318.00 ± 76.00 ^c^	151.00 ± 36.00 ^h^	60.70 ± 41.60 ^a^
B6	67.00 ± 9.40 ^a^	58.20 ± 8.10 ^fg^	48.20 ± 6.70 ^b^	56.10 ± 7.90 ^fg^	90.40 ± 12.70 ^c^	71.40 ± 10.00 ^f^	134.00 ± 19.00 ^d^	53.60 ± 7.50 ^g^	39.00 ± 5.50 ^b^
B9	19.49 ± 3.40 ^a^	21.23 ± 2.54 ^f^	22.25 ± 0.31 ^a^	9.36 ± 0.63 ^g,^*	67.62 ± 7.78 ^b^	34.30 ± 0.92 ^h,^*	26.02 ± 5.12 ^a^	21.08 ± 1.30 ^f^	26.31 ± 4.18 ^a^
Minerals (mg/100 g)									
Calcium	55.00 ± 11.00 ^a^	43.00 ± 8.60 ^f^	79.00 ± 16.00 ^a^	77.00 ± 15.00 ^g^	72.00 ± 14.00 ^a^	52.00 ± 10.00 ^f^	33.00 ± 6.60 ^b^	27.00 ± 5.40 ^h^	120.00 ± 24.00 ^c^
Copper	0.27 ± 0.05 ^a^	0.29 ± 0.06 ^f^	0.22 ± 0.04 ^a^	0.26 ± 0.05 ^f^	0.24 ± 0.05 ^a^	0.24 ± 0.06 ^f^	0.21 ± 0.04 ^a^	0.18 ± 0.04 ^f^	0.19 ± 0.04 ^a^
Iron	2.30 ± 0.46 ^a^	1.80 ± 0.04 ^f,^*	1.60 ± 0.32 ^a^	1.50 ± 0.30 ^f^	1.30 ± 0.26 ^a^	1.30 ± 0.26 ^f^	2.00 ± 0.40 ^a^	1.50 ± 0.30 ^f^	1.70 ± 0.34 ^a^
Magnesium	39.00 ± 7.80 ^a^	26.00 ± 5.20 ^f,^*	37.00 ± 7.40 ^a^	31.00 ± 6.20 ^f^	44.00 ± 8.80 ^a^	28.00 ± 5.60 ^f,^*	32.00 ± 6.40 ^a^	13.00 ± 2.60 ^f^	33.00 ± 6.60 ^a^
Manganese	0.44 ± 0.09 ^a^	0.33 ± 0.07 ^f^	0.55 ± 0.11 ^ab^	0.50 ± 0.10 ^g^	0.86 ± 0.17 ^c^	0.67 ± 0.13 ^g^	0.38 ± 0.08 ^a^	0.26 ± 0.05 ^f^	0.80 ± 0.16 ^bc^
Phosphorus	150.00 ± 30.00 ^a^	110.00 ± 22.00 ^f^	120.00 ± 24.00 ^a^	110.00 ± 22.00 ^f^	140.00 ± 28.00 ^a^	97.00 ± 19.00 ^f^	140.00 ± 28.00 ^a^	53.00 ± 11.00 ^f^	110.00 ± 22.00 ^a^
Potassium	300.00 ± 60.00 ^a^	250.00 ± 50.00 ^f^	230.00 ± 46.00 ^ab^	230.00 ± 46.00 ^f^	170.00 ± 34.00 ^b^	140.00 ± 28.00 ^g^	230.00 ± 46.00 ^ab^	81.00 ± 16.00 ^h^	260.00 ± 52.00 ^a^
Zinc	0.94 ± 0.19 ^a^	0.77 ± 0.16 ^f^	0.76 ± 0.16 ^a^	0.74 ± 0.15 ^f^	1.10 ± 0.22 ^a^	1.10 ± 0.22 ^f^	0.99 ± 0.20 ^a^	0.48 ± 0.10 ^g^	0.67 ± 0.14 ^a^
Bioactive/Antinutritional compounds (mg/100 g)									
Phytic acid	627.33 ± 42.35 ^ad^	386.32 ± 25.46 ^f,^*	532.43 ± 28.28 ^b^	470.31 ± 48.05 ^gh^	693.94 ± 40.67 ^acd^	526.40 ± 22.26 ^g,^*	714.46 ± 28.85 ^cd^	412.59 ± 36.26 ^fh^	683.66 ± 65.66 ^d^
Saponins	106.02 ± 4.11 ^a^	118.60 ± 4.84 ^f^	103.95 ± 5.69 ^a^	107.58 ± 0.55 ^g^	121.86 ± 4.68 ^b^	116.84 ± 0.22 ^f,^*	174.73 ± 2.66 ^c^	98.73 ± 2.72 ^h^	122.12 ± 2.33 ^b^
Tannins	6.78 ± 0.36 ^a^	5.44 ± 0.27 ^f,^*	1.69 ± 0.04 ^b^	1.82 ± 0.11 ^g^	16.61 ± 1.89 ^c^	13.67 ± 1.48 ^h^	12.53 ± 0.18 ^d^	7.54 ± 0.08 ^i^	2.24 ± 0.10 ^e^
Total polyphenols	66.34 ± 2.45 ^a^	67.17 ± 2.48 ^f^	15.00 ± 1.08 ^b^	18.66 ± 1.34 ^g^	24.98 ± 1.15 ^c^	27.59 ± 1.27 ^h^	284.32 ± 3.26 ^d^	71.84 ± 0.82 ^i^	15.77 ± 0.75 ^b^
Dry matter (g/100 g)	34.05	31.00	30.02	28.20	38.09	33.80	34.28	24.20	33.58

AA: amino acids. Data are means ± SD (*n* = 3–4). Means values on the same line and for a same cooking method followed by a different superscript letter are significantly different (*p* < 0.05): a, b, c, d, e are used to compare household-cooked pulses; f, g, h, i are used to compare canned pulses. An asterisk indicates a significant difference (*p* < 0.05) between the two cooking methods (highlighted in gray in the Table). Vitamin C, iodine, and selenium were not detected in any samples. n.d. = not detectable or below the limit of quantitation in the sample.

**Table 2:** Amino acid content of prepared kidney beans, white beans, chickpeas, green and brown lentils, and flageolets.

Pulses	Red Beans		White Beans		Chickpeas		Green Lentils	Brown Lentils	Flageolets
Cooking Method	Household cooking	Canning	Household cooking	Canning	Household cooking	Canning	Household cooking	Canning	Household cooking
Essential AA (g/100g)									
Cys	0.095 ± 0.008 ^ab^	0.090 ± 0.016 ^f^	0.087 ± 0.008 ^ab^	0.083 ± 0.014 ^f^	0.081 ± 0.007 ^bc^	0.075 ± 0.013 ^f^	0.064 ± 0.006 ^c^	0.072 ± 0.012 ^f^	0.114 ± 0.010 ^ab^
His	0.279 ± 0.004 ^a^	0.184 ± 0.005 ^f,^*	0.203 ± 0.003 ^b^	0.151 ± 0.004 ^g,^*	0.214 ± 0.003 ^bc^	0.177 ± 0.005 ^f,^*	0.222 ± 0.003 ^c^	0.129 ± 0.004 ^h^	0.190 ± 0.003 ^d^
Ile	0.458 ± 0.008 ^a^	0.346 ± 0.012 ^f,^*	0.363 ± 0.006 ^bc^	0.234 ± 0.008 ^g,^*	0.379 ± 0.007 ^b^	0.307 ± 0.011 ^h,^*	0.355 ± 0.006 ^c^	0.201 ± 0.007 ^i^	0.333 ± 0.006 ^d^
Leu	0.785 ± 0.007 ^a^	0.589 ± 0.011 ^f,^*	0.632 ± 0.006 ^b^	0.459 ± 0.009 ^g,^*	0.635 ± 0.006 ^b^	0.535 ± 0.010 ^h,^*	0.620 ± 0.006 ^b^	0.394 ± 0.007 ^i^	0.594 ± 0.006 ^c^
Lys	0.670± 0.011 ^a^	0.437 ± 0.005 ^f,^*	0.487 ± 0.008 ^b^	0.412 ± 0.005 ^g,^*	0.521 ± 0.008 ^c^	0.426 ± 0.005 ^fg,^*	0.576 ± 0.009 ^d^	0.354 ± 0.004 ^h^	0.475 ± 0.008 ^b^
Met	0.105 ± 0.014 ^a^	0.067 ± 0.013 ^f,^*	0.086 ± 0.012 ^b^	0.059 ± 0.011 ^f,^*	0.087 ± 0.012 ^b^	0.075 ± 0.014 ^f^	0.040 ± 0.005 ^c^	0.051 ± 0.010 ^f^	0.098 ± 0.013 ^ab^
Phe	0.572 ± 0.013 ^a^	0.400 ± 0.008 ^f,^*	0.429 ± 0.010 ^bd^	0.320 ± 0.006 ^g,^*	0.476 ± 0.011 ^c^	0.414 ± 0.008 ^f,^*	0.441 ± 0.010 ^b^	0.275 ± 0.005 ^h^	0.410 ± 0.009 ^d^
Thr	0.457 ± 0.006 ^a^	0.316 ± 0.003 ^f,^*	0.340 ± 0.005 ^b^	0.253 ± 0.003 ^g,^*	0.364 ± 0.005 ^c^	0.299 ± 0.003 ^h,^*	0.349 ± 0.005 ^b^	0.217 ± 0.002 ^i^	0.348 ± 0.005 ^b^
Trp	0.110 ± 0.034 ^ac^	0.071 ± 0.020 ^f^	0.066 ± 0.020 ^abc^	0.061 ± 0.017 ^f^	0.037 ± 0.011 ^b^	0.041 ± 0.012 ^f^	0.064 ± 0.019 ^c^	0.052 ± 0.015 ^f^	0.086 ± 0.026 ^c^
Tyr	0.425 ± 0.009 ^a^	0.295 ± 0.007 ^f,^*	0.314 ± 0.007 ^b^	0.215 ± 0.005 ^g,^*	0.280 ± 0.006 ^c^	0.241 ± 0.005 ^h,^*	0.301 ± 0.007 ^bc^	0.185 ± 0.004 ^i^	0.298 ± 0.006 ^c^
Val	0.512 ± 0.002 ^a^	0.384 ± 0.010 ^f,^*	0.407 ± 0.002 ^b^	0.263 ± 0.007 ^g,^*	0.379 ± 0.002 ^c^	0.303 ± 0.008 ^h,^*	0.340 ± 0.002 ^d^	0.226 ± 0.006 ^i^	0.387 ± 0.002 ^e^
Total essential AA	4.469	3.180	3.415	2.509	3.453	2.891	3.429	2.153	3.332
Non-essential AA (g/100g)									
Ala	0.409 ± 0.003 ^a^	0.280 ± 0.006 ^fg,^*	0.305 ± 0.002 ^b^	0.262 ± 0.006 ^f,^*	0.350 ± 0.003 ^c^	0.286 ± 0.006 ^g,^*	0.363 ± 0.003 ^d^	0.225 ± 0.005 ^h^	0.300 ± 0.002 ^b^
Arg	0.581 ± 0.015 ^a^	0.375 ± 0.011 ^f,^*	0.403 ± 0.010 ^b^	0.477 ± 0.014 ^g,^*	0.629 ± 0.016 ^c^	0.514 ± 0.015 ^h,^*	0.721 ± 0.018 ^d^	0.409 ± 0.012 ^i^	0.391 ± 0.010 ^e^
Asp ^¤^	1.170± 0.014 ^a^	0.807 ± 0.015 ^f,^*	0.858 ± 0.010 ^b^	0.713 ± 0.014 ^g,^*	0.946 ± 0.011 ^c^	0.775 ± 0.015 ^f,^*	1.022 ± 0.012 ^d^	0.612 ± 0.012 ^h^	0.822 ± 0.001 ^e^
Glu ^¤¤^	1.555 ± 0.018 ^a^	1.059 ± 0.024 ^f,^*	1.122 ± 0.013 ^b^	1.041 ± 0.024 ^f,^*	1.297 ± 0.015 ^c^	1.054 ± 0.024 ^f,^*	1.522 ± 0.018 ^d^	0.894 ± 0.020 ^g^	0.996 ± 0.012 ^e^
Gly	0.381 ± 0.003 ^a^	0.267 ± 0.005 ^f,^*	0.287 ± 0.002 ^b^	0.246 ± 0.005 ^g,^*	0.320 ± 0.003 ^c^	0.263 ± 0.005 ^f,^*	0.349 ± 0.003 ^d^	0.211 ± 0.004 ^h^	0.290 ± 0.002 ^b^
Pro	0.476 ± 0.013 ^a^	0.333 ± 0.012 ^f,^*	0.355 ± 0.010 ^b^	0.331 ± 0.012 ^f,^*	0.389 ± 0.011 ^c^	0.325 ± 0.012 ^f,^*	0.443 ± 0.012 ^a^	0.284 ± 0.011 ^g^	0.350 ± 0.010 ^b^
Ser	0.629 ± 0.003 ^a^	0.434 ± 0.011 ^f,^*	0.459 ± 0.002 ^b^	0.339 ± 0.008 ^g,^*	0.437 ± 0.002 ^c^	0.355 ± 0.000 ^h,^*	0.464 ± 0.002 ^b^	0.291 ± 0.007 ^i^	0.440 ± 0.002 ^c^
Total non-essential AA	5.205	3.556	3.787	3.410	4.367	3.571	4.883	2.926	3.588

AA: amino acids. ^¤^ Asp = asparagine + aspartic acid; ^¤¤^ Glu = glutamine + glutamic acid. Data are means ± SD (*n* = 3). Mean values on the same line and for a same cooking method followed by a different superscript letter are significantly different (*p* < 0.05): a, b, c, d, e are used to compare household-cooked pulses; f, g, h, i are used to compare canned pulses. An asterisk indicates a significant difference (*p* < 0.05) between the two cooking methods (highlighted in gray in the Table).
